# Development and validation of a quantitative PCR for the detection of Guinea worm (*Dracunculus medinensis*)

**DOI:** 10.1371/journal.pntd.0010830

**Published:** 2022-10-07

**Authors:** Sarah M. Coker, Erin K. Box, Natalie Stilwell, Elizabeth A. Thiele, James A. Cotton, Ellen Haynes, Michael J. Yabsley, Christopher A. Cleveland

**Affiliations:** 1 Southeastern Cooperative Wildlife Disease Study, Department of Population Health, College of Veterinary Medicine, University of Georgia, Athens, Georgia, United States of America; 2 Department of Pathobiology and Population Medicine, College of Veterinary Medicine, Mississippi State University, Starkville, Mississippi, United States of America; 3 Department of Biology, Vassar College, Poughkeepsie, New York, United States of America; 4 Wellcome Sanger Institute, Wellcome Genome Campus, Hinxton, Cambridgeshire, United Kingdom; 5 Warnell School of Forestry and Natural Resources, University of Georgia, Athens, Georgia, United States of America; 6 Center for Ecology of Infectious Diseases, University of Georgia, Athens, Georgia, United States of America; Federation University Australia, AUSTRALIA

## Abstract

*Dracunculus medinensis* (Guinea worm) is a parasitic nematode that can cause the debilitating disease dracunculiasis (Guinea worm disease) in humans. The global Guinea Worm Eradication Program has led intervention and eradication efforts since the 1980s, and Guinea worm infections in people have decreased >99.99%. With the final goal of eradication drawing nearer, reports of animal infections from some remaining endemic countries pose unique challenges. Currently, confirmation of suspected Guinea worm infection relies on conventional molecular techniques such as polymerase chain reaction (PCR), which is not specific to Guinea worm and, therefore, requires sequencing of the PCR products to confirm the identity of suspect samples, a process that often takes a few weeks. To decrease the time required for species confirmation, we developed a quantitative PCR assay targeting the mitochondrial cytochrome *b* (*cytb*) gene of Guinea worm. Our assay has a limit of detection of 10 copies per reaction. The mean analytical parameters (± SE) were as follows: efficiency = 93.4 ± 7.7%, *y*-intercept = 40.93 ± 1.11, slope = -3.4896 ± 0.12, and the R^2^ = 0.999 ± 0.004. The assay did not amplify other nematodes found in Guinea worm-endemic regions and demonstrated 100% diagnostic sensitivity and specificity. Implementation of this quantitative PCR assay for Guinea worm identification could eliminate the need for DNA sequencing to confirm species. Thus, this approach can be implemented to provide more rapid confirmation of Guinea worm infections, leading to faster execution of Guinea worm interventions while increasing our understanding of infection patterns.

## Author summary

Guinea worm (*Dracunculus medinensis*) is a parasitic nematode that causes debilitating disease in humans. The Guinea Worm Eradication Program would benefit from having a rapid molecular test that can confirm species identification without time-consuming DNA sequencing. We developed a qPCR protocol targeting the mitochondrial cytochrome *b* (*cytb)* gene of Guinea worm. The assay was validated analytically over 12 experiments using a standard serial dilution as well as diagnostically on DNA samples from non-target host species and other parasites (n = 180) and Guinea worm samples (n = 200) from a diversity of hosts and geographic regions. This assay could reliably detect 10 copies of the target DNA sequence and had a mean efficiency of 93.4% with 100% diagnostic sensitivity and specificity.

## Introduction

*Dracunculus medinensis* (Guinea worm; Family Dracunculidae, Order Spirurida) is a parasitic nematode that causes the debilitating disease dracunculiasis (Guinea worm disease) in humans. Dracunculiasis is caused by the development and emergence of a large, gravid female worm measuring up to a meter long [1,2]. In particular, the emergence of the gravid female through the skin of a distal extremity can cause considerable pain for several weeks and can leave the affected individual with an open wound that is susceptible to secondary bacterial infections [1,2]. The Guinea Worm Eradication Program (GWEP) has worked to eradicate this parasite since the 1980s, during which time human infections have decreased dramatically from an estimated 3.5 million cases in 1986 to just 15 in 2021 [1,3,4].

*Dracunculus* transmission occurs when an obligate intermediate host, a cyclopoid copepod, infected with third-stage larvae [L3s] is ingested by a susceptible definitive host [2,5]. As measures were implemented to minimize the transmission of Guinea worm (primarily focusing on ensuring access to clean drinking water free of infected copepods), dracunculiasis cases decreased dramatically [1,3,6,7]. However, in recent years, infections in animals have complicated eradication efforts; most animal infections have occurred in domestic dogs (*Canis lupus familiaris*) in Chad, Africa, with fewer numbers of dogs in other countries, domestic cats (*Felis catus*) in Chad, and baboons (*Papio* spp.) in Ethiopia [8]. Accordingly, while traditional Guinea worm control measures remain in place, new measures focusing on preventing transmission to animals have been enacted to address this final barrier to eradication. One vital aspect of these control measures is rapid containment of infected animal hosts and confirmation of the identity of any emerging worms suspected to be Guinea worm.

Accurate surveillance and effective implementation of some intervention measures rely on confirmation that suspected infections are actually *D*. *medinensis*. However, expeditious molecular confirmation of suspected nematodes as Guinea worm can be challenging. Currently, samples are processed via a conventional PCR that is not specific to Guinea worm, amplifying other nematode DNA; therefore, this PCR must be followed by Sanger sequencing in order to confirm the identity. While this method reliably differentiates Guinea worm from other nematodes, the turnaround time from sample receipt at appropriate diagnostic laboratories to species confirmation can take up to two weeks. Therefore, the purpose of this Guinea worm quantitative polymerase chain reaction assay (GW qPCR) is to provide a highly sensitive and specific diagnostic tool for rapid identification of suspect infections with adult, female Guinea worms in humans and domestic and wild animals. This GW qPCR platform avoids the need for sequencing and can produce results (including the time needed for DNA extraction) within 24 hours.

## Methods

The development and validation of the GW qPCR protocol were carried out at the Southeastern Cooperative Wildlife Disease Study (SCWDS) at the University of Georgia in Athens, Georgia, USA. First, primer and probe sets were designed to target Guinea worm based on closely related nematode genomic data in the National Center for Biotechnology Information (NCBI) database. Second, the conditions for the GW qPCR were optimized using a synthetic Guinea worm amplicon serially diluted as a standard curve along with extracted DNA from Guinea worms as positive samples and DNA extracts from the related *Dracunculus insignis* as negative control samples. Next, the analytical sensitivity was estimated using standard curve C_q_ values generated in-house (n = 9) and at other labs (n = 3). Last, the diagnostic sensitivity (D_Sn_) and specificity (D_Sp_) were estimated with non-target samples (n = 180) and Guinea worm samples (n = 200) run in triplicate.

### *In silico* qPCR primer and probe design

Available mitochondrial genome sequences for Guinea worm were downloaded from GenBank. Sequences for close phylogenetic relatives (*D*. *insignis* and *D*. *lutrae*) were generated by assembling the Illumina sequencing reads for these species from [9] using v1.9 of the mitoBIM pipeline [10] with mira v4.0.2 [11], run for 30 iterations and a baiting kmer length of 41, in ‘quick’ mode using the reference mitochondrial genome for Guinea worm [9] as bait sequence. Assembled mitochondrial sequences were then annotated using Prokka v1.14.5 [12], and the ORF with the highest scoring blastp hit with e-value < = 0.01 when compared against each *O*. *volvulus* mitochondrial protein set [13] was annotated as the likely homolog for that sample. For Guinea worm, four mitochondrial genes (cytochrome *c* oxidase subunit 1 [*COI*], cytochrome *c* oxidase subunit 3 [*cox3*], cytochrome *b* [*cytb*], and NADH dehydrogenase 3 [*nd3*]) had significant coverage in GenBank and were identified as candidate genes for primer and probe design [14]. For each gene, sequences were aligned using the MUSCLE alignment feature [15] in Geneious Prime (Ver 2019.0.4). The consensus sequences for each gene alignment were generated at 100% identity threshold, revealing homologous regions within the *cytb* gene that were suitable candidates for primer and probe design. A homologous region in the *cox3* gene was also identified (see S1 Methods).

The consensus sequence generated from all available Guinea worm *cytb* gene sequences was imported into PrimerQuest software (Integrated DNA Technologies, Coralville, IA; Table 1) for primer and hydrolysis probe design. Primer and probe sequences were then aligned with *cytb* consensus sequences from the other nematode taxa to confirm mismatches. Thus, the *in silico* specificity of the primer and probe combination was limited to Guinea worm. Primers and probe were synthesized by MilliporeSigma (Burlington, MA, USA).

**Table 1:** Primers and Taqman probe targeting the mitochondrial cytochrome *b* (*cytb*) gene of Guinea worm (*Dracunculus medinensis)* for qPCR amplification and development of a gBlock standard.

Primer	Sequence (5’→3’)	Gene Position (Guinea worm)	Length (bases)	Est. Tm (°C)*	GC Content (%)	Amplicon Size (bp)
F	ATGGTTGGTTGTATCGT	206–223	17	56, 53	41.2	124
R	CCCTCAACCAAACCAAA	329–312	17	57, 59.5	47.1	
P	FAM-AGGGTTTGTTTAATAGAAGGTATCGTCTTT-BHQ	281–311	30	65, 65.4	33	

*Two melting temperature estimates are provided. The first was calculated during the assay design by the PrimerQuest software. The second was provided by the manufacturer (MilliporeSigma).

### Standard curve production and estimation of qPCR assay parameters

To develop a standard curve, a gBlock Gene Fragment (Integrated DNA Technologies, Iowa, USA) was generated using a known Guinea worm *cytb* gene target sequence plus a 50-bp overhang on both ends of the target sequence, resulting in a 224-bp sequence for positions 156–379. Due to the low GC content, adaptor sequences were added to both ends of the *cytb* gene fragment to allow for successful synthesis, resulting in a final gene fragment length of 283-bp. Standard curves were generated using 10-fold serial dilutions and tested in triplicate at SCWDS to estimate the efficiency [10–1/slope—1; 16], repeatability, and limit of detection (LOD).

The 10-fold serial dilutions of the gBlock (ranging from 1 x 10^8^ copies to 1 copy per reaction) were run in triplicate to optimize the qPCR protocol (e.g., determining the most efficient annealing temperature at 55.1°C) and estimate the analytical sensitivity and specificity of the assay. A total of 12 standard curve experiments were performed to estimate the analytical efficiency, slope, *y*-intercept, correlation coefficient (R^2^), LOD/analytical sensitivity, repeatability, and reproducibility. Three of these 12 experiments were performed by labs at the University of Georgia that were not associated with this project. The LOD was defined as the dilution with the lowest copy number detected 50% of the time [17]. Repeatability and reproducibility were calculated as the percent coefficient of variation using the mean and standard deviation (SD) of C_q_ values within or among, respectively, the experiments.

### Sample acquisition

Gravid female Guinea worms preserved in 70% ethanol that were morphologically and genetically confirmed as Guinea worm were obtained from Guinea worm endemic countries in Africa from 2006–2020 [Table 2; 9,14]. Sources of these samples represented the full extent of the currently known definitive non-human hosts (dog, cat, baboon, and leopard) and extant geographic range (Angola, Cameroon, Chad, Ethiopia, Mali, and South Sudan) of GW and thus sufficiently represent the samples on which this assay will be used [18]. DNA extracts from sections of the worms containing larvae, or aliquots of larvae expressed from adult females after collection, were used to optimize and validate this protocol. Host tissue and additional parasite samples were obtained through multiple sources to test assay specificity. Intermediate [i.e., cyclopoid copepods; 1,5,19], potential paratenic and transport [20–23], and definitive host species [i.e., domestic cat, domestic dog, and baboon; 5,14,24] were included (Table 3). Morphologically-identified and molecularly-confirmed nematodes (n = 91) and one trematode species were obtained from research samples or wildlife submitted for diagnostic evaluation by the SCWDS diagnostic service (Table 4). These non-target samples included other dracunculids (n = 62) that are most likely to cross-react in the assay [14,25,26]. *Dracunculus insignis* samples were primarily obtained from raccoons, a common host of this species in North America, with additional samples from mustelids and skunks [27–31]. Two adult, female *Dracunculus ophidensis* [32] from Lake Erie watersnakes (*Nerodia sipedon insularum*) in the USA were available; these nematodes were presumed to be *D*. *ophidensis*, as no males, which are necessary to morphologically identify species in this genus [32–35], were available for morphological confirmation, and minimal genetic information was available on Genbank for comparison at the time of this validation. Other non-target parasites were obtained from potential Guinea worm hosts.

**Table 2:** Summary of Guinea worm (*Dracunculus medinensis*) samples used to estimate diagnostic sensitivity and specificity showing the host species, location, and year of origin.

Host Species	Location	Year	Number of GW samples
Domestic dog (*Canis familiaris)*	Angola	2019	3^⧫^
	Chad	2020	151^⧫^
		2019	2
		2018	1
	Ethiopia	2014	1
		2017	1
		2018	5
	Mali	2018	6
		2019	2^⧫^
Domestic cat (*Felis catus)*	Chad	2020	1
	Mali	2018	1
Human *(Homo sapiens sapiens)*	Angola	2019	1
	Cameroon	2019	1
	Côte d’Ivoire	2006	1
	Ethiopia	2013	2
	Mali	2015	1
	South Sudan	2013	3
		2014	5
		2016	1
		2018	4
		2019	1
Domestic ferret (*Mustela putorius furo)*	Chad/USA*	2019	3
Leopard (*Panthera pardus pardus)*	Ethiopia	2019	2^⧫^
Olive baboon (*Papio anubis)*	Ethiopia	2013	1
			N = 200

*Indicates samples from experimental infections conducted at the University of Georgia (UGA), Athens, Georgia, USA, using larvae of adult worms from Chad.

^⧫^Indicates groups/samples used for qPCR assay validation at other laboratories at UGA. See [9] and [14] for source information.

**Table 3:** Summary of non-target host samples used to estimate diagnostic sensitivity and specificity showing host phylum, common name, scientific name, location of origin, and number of samples.

Phylum	Host	Order:Family	Location	No.	Source Citation
Arthropoda	Copepod	*Mesocyclops* sp. (Cyclopoida:Cyclopidae)	Chad	9^⧫^	[36]
	Copepod	*Thermocyclops decipiens* (Cyclopoida:Cyclopidae)	Chad	1	[36]
	Copepod	*Macrocyclops* sp. (Cyclopoida:Cyclopidae)	USA	5	[36]
Chordata	Crowned bullfrog	*Hoplobatrachus occipitalis* (Anura:Dicroglossidae)	Chad	17^⧫^	[36]
	Grass frog	*Ptychadena* sp. (Anura:Ptychadena)	Chad	9	[36]
	Galam white-lipped frog	*Amnirana galamenis* (Anura:Ranidae)	Chad	1	[36]
	Domestic dog	*Canis familiaris* (Carnivora:Canidae)	Chad	33	[37]
	Domestic cat	*Felis catus* (Carnivora:Felidae)	USA	1	⬢
	North American river otter	*Lontra canadensis* (Carnivora:Mustelidae)	USA	2	⬢
	Olive baboon	*Papio anubis* (Primates:Cercopithecidae)	Chad	1^⧫^	[14]
	African sharptooth catfish	*Clarias gariepinus* (Siluriformes:Clariidae)	Chad	7	[36]
	Catfish	*Synodontis* sp. (Siluriformes:Mochokidae)	Chad	1	[36]
	Common agama	*Agama agama* (Squamata:Agamidae)	Chad	1	[36]
				N = 88	

^⧫^Indicates groups/samples used for qPCR validation at other laboratories at UGA.

^⬢^ Submitted to the SCWDS diagnostic service, Animal Use Protocol: A2020 11-010-Y2-A3.

**Table 4:** Summary of non-target parasite samples used to estimate diagnostic sensitivity and specificity showing taxonomic grouping, location of origin, and number of samples.

Phylum	Family	Parasite	Host Order:Family	Location	No.	Source Citation
Nematoda	Dracunculidae	*Anguillicoloides crassus*	Anguilliformes:Anguillidae	USA	2	[25]
	Camallanidae	*Batrachocamallanus xenopodis*	Characiformes:Alestidae^▲^	Chad	1	[22]
	Ascarididae	*Baylisascaris procyonis*	Rodentia:Sciuridae	USA	1	[25]
	Onchocercidae	*Brugia pahangi*	Carnivora:Canidae	USA	1	⬢
	Camallanidae	ND	Characiformes:Alestidae^▲^	Chad	2	[36]
	Onchocercidae	*Dirofilaria immitis*	Carnivora:Canidae	USA	1	⬢
	Onchocercidae	*Dirofilaria lutrae*	Carnivora:Mustelidae	USA	3	[38]
	Dracunculidae	*Dracunculus insignis*	Carnivora:Mephitidae	USA	1	⬢
			Carnivora:Mustelidae		22	⬢
			Carnivora:Procyonidae		24^⧫^	[25]
			Didelphimorphia: Didelphidae		9^⧫^	[25]
	Dracunculidae	*Dracunculus ophidensis* (presumptive)	Squamata:Colubridae	USA	2	⬢
	Dracunculidae	*Dracunculus* sp.	Carnivora:Mustelidae	USA	1	[25]
			Didelphimorphia: Didelphidae		1	[25]
	Onchocercidae	ND	Primates:Cercopithecidae^¶^	Chad	1	[9,14]
			Siluriformes:Bagridae^▲^		1	[36]
	Philometridae	ND	ND:ND	USA	1	⬢
	Filariidae	*Filaria taxidae*	Carnivora:Mustelidae	USA	1	[39]
	Gongylonematidae	*Gongylonema pulchrum*	Carnivora:Ursidae	USA	1	⬢
	Mermithidae	ND	ND:ND	Chad	4^⧫^	[9,14]
	Onchocercidae	*Ochoterenella* sp.	Squamata:Varanidae^▲^	Chad	1	[36]
	Philometridae	*Philometra charlestoni*	Perciformes:Serranidae	USA	1	⬢
	Philometridae	*Philometroides paralichthydis*	Pleuronectiformes: Paralichthyidae	USA	1	⬢
	Anisakidae	*Pseudoterranova* sp.	Urodela:ND	USA	4	⬢
	Thelaziidae	*Rhabdochona* sp.	Cichliformes:Cichlidae^▲^	Chad	1	[36]
			Siluriformes:Schilbeidae^▲^	Chad	1	
	Diplotriaenidae	*Serratospiculum* sp.	Aves	Chad	1	[9,14]
	Gnathostomatidae	*Spiroxys* sp.	Cichliformes:Cichlidae^▲^	Chad	1	[36]
Platyhelminthes	Class:Trematoda	ND	Cichliformes:Cichlidae^▲^	Chad	1	[36]
					N = 92	

“ND” denotes where no data was available.

^⧫^Indicates groups/samples used for validation at other laboratories at UGA.

^▲^Indicates potential paratenic/transport hosts of Guinea worm.

^¶^Indicates potential definitive hosts of Guinea worm.

^⬢^ Submitted to the SCWDS diagnostic service, Animal Use Protocol: A2020 11-010-Y2-A3.

### Ethical clearance

All samples included in this research were derived from materials collected either under procedures sanctioned by the World Health Organization and national governments for containment and treatment of GW, or were collected under authorizations for research or diagnostic services. These authorizations are granted by Institutional Animal Care and Use Committees, which provide oversight of each Animal Use Protocol (AUP). Some of the samples collected for the purpose of research and diagnostics have not been included in published reports prior to this manuscript.

### Extraction of DNA from tissue samples

Host and parasite tissues were preserved and stored either frozen at -20°C or preserved in 70–100% ethanol at ambient temperature. Sections of worms, portions of tissues, or whole larvae in ethanol were incubated at room temperature in 1.5 mL microcentrifuge tubes for at least 12 hours to allow residual ethanol to evaporate. Frozen worm or tissue samples were thawed and portions removed for DNA extraction. All sample DNA extractions conducted at SCWDS for this study were carried out with the DNeasy Blood and Tissue Extraction Kit (Qiagen, Valencia, CA, USA) using the manufacturer’s protocol. DNA extracts were stored at -20°C.

### Detection of Guinea worm DNA using qPCR

The total reaction volume of qPCR reactions was 15 μl, with 7.5 μl Taq-Man Universal PCR Master Mix 2× (Applied Biosystems, Thermo Fisher Scientific, Inc., Waltham, MA, USA), 1 μl detection enhancer (Applied Biosystems), 333 nM of the forward primer and probe, 666 nM of the reverse primer, and 2.5 μl of the sample/standard. Reactions were run on a CFX96 touch Real Time Detection System (BioRad, Hercules, CA, USA). Each run was performed using white Hard Shell plates (BioRad) with optically clear PCR plate sealing adhesive film (BioRad) and included samples, a standard curve, and NTC (No Template Control, to which molecular grade water was added instead of DNA). All samples and controls were tested in triplicate. The thermocycling conditions were 50°C for 2 min, 95°C for 10 min, followed by 40 cycles of amplification at 95°C for 15 sec, and 55.1°C for 1 min.

### Estimation of diagnostic sensitivity and specificity

Guinea worm (n = 200), host (n = 88), and non-target parasite (n = 92) samples (Tables 2–4) were tested in triplicate to assess the diagnostic sensitivity and specificity of the GW qPCR assay. A sample was considered “positive” when the C_q_ values for all three replicates surpassed the detection threshold at ≤ 40 cycles, “suspect” when only two samples were above the detection threshold, and “negative” when one or none of the replicates was above the threshold. “Suspect” samples were retested in triplicate to confirm status.

To ensure the protocol was sufficiently optimized for various laboratory settings, each of the three external labs that participated in the analytical sensitivity/specificity experiments also tested 17 blind DNA samples in triplicate (source groups indicated with ⧫ in Tables 2–4).

## Results

### *In silico* qPCR primer and probe design

Few mitochondrial sequences from close genetic relatives of Guinea worm were available for *in silico* specificity analysis. No *cytb* gene sequences for *D*. *insignis* and *D*. *lutrae* are currently available in GenBank; we thus generated mitochondrial genome sequences from 18 *D*. *insignis* and one *D*. *lutrae* Illumina sequencing libraries derived from 6 distinct biological samples. Thus, we generated Illumina sequencing libraries from 5 *D*. *insignis* and one *D*. *lutrae*.

**Fig 1:**
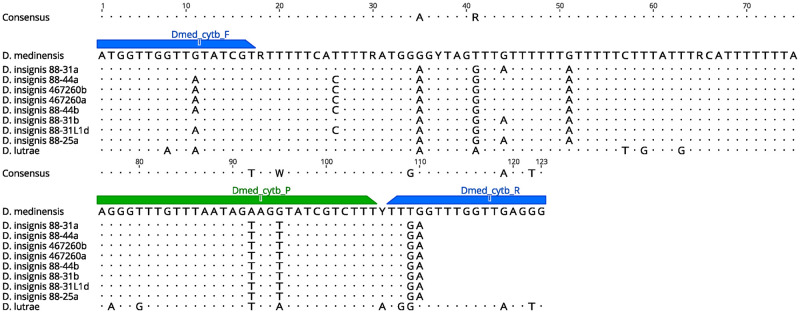
Alignment of partial (123-bp) *cytb* gene for three *Dracunculus* species demonstrating the *in silico* specificity of the qPCR primers (Dmed_cytb_F/R) and probe (Dmed_cytb_P).

For the designed *cytb* primer/probe set, three of the available *D*. *insignis* sequences showed complete homology to the *cytb* forward primer but not the reverse primer or probe (Fig 1). Initial optimization and specificity testing of the *cytb* gene resulted in nonspecific amplification of *D*. *insignis* samples, which was resolved by increasing the annealing temperature and adding detection enhancer to the qPCR reaction mix.

### Standard curve production and estimation of qPCR assay analytical parameters

**Fig 2:**
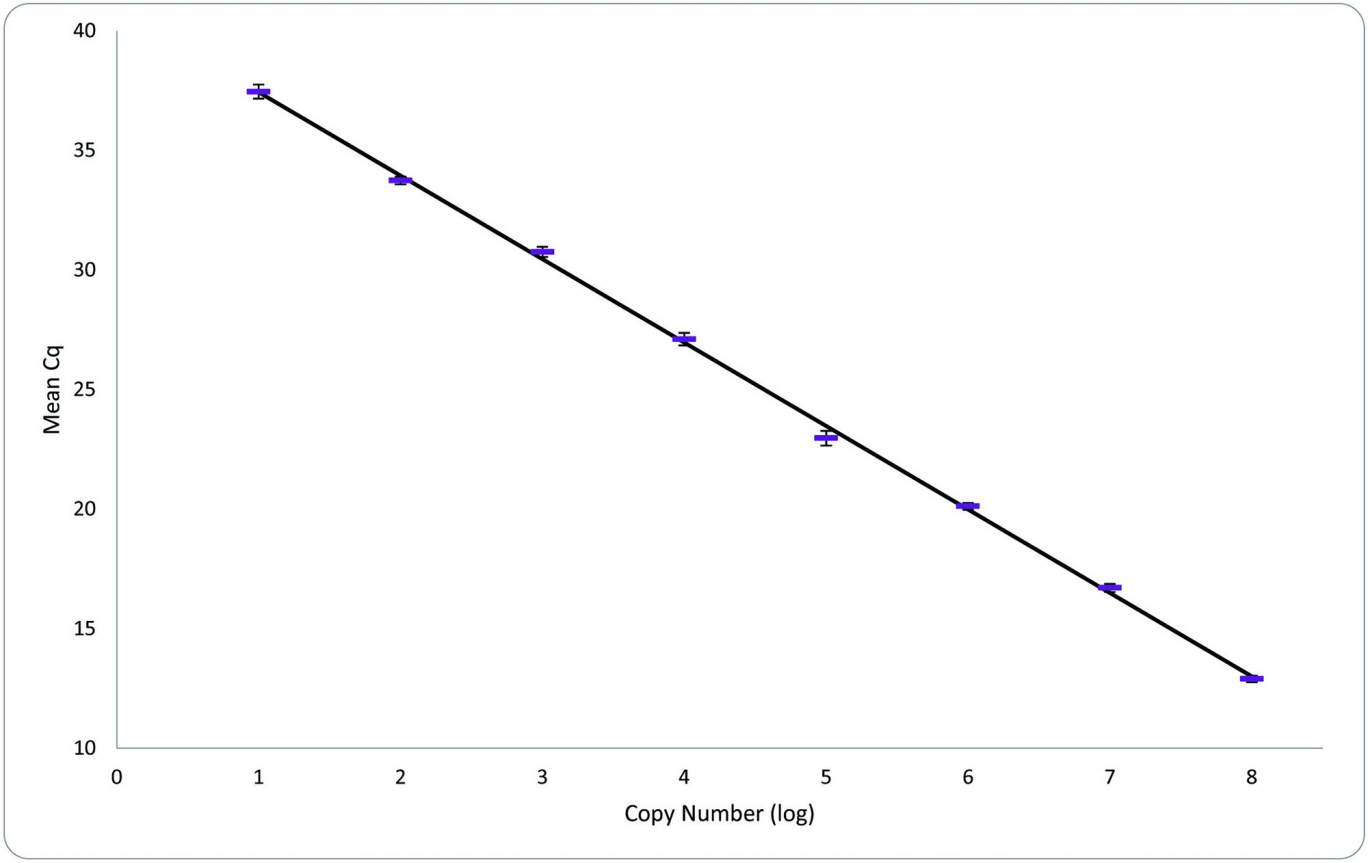
Guinea worm (*Dracunculus medinensis*) qPCR standard curve generated using 12 sets of 10-fold dilutions of plasmid run in triplicate. The x-axis is the log copy number of the plasmid at each dilution, and the y-axis is the C_q_ value. The mean C_q_ values (± SE) are plotted.

The LOD was determined to be 10 copies per reaction, which was detected in 86% (31/36 replicates) of the reactions throughout the experiments (n = 12; Table 5); detection of 1 copy per reaction was <50%. Average (± SE) parameters over 12 experiments were as follows: efficiency = 93.4 ± 7.7%, *y*-intercept = 40.93 ± 1.11, slope = -3.4896 ± 0.12, and the R^2^ = 0.999 ± 0.004 (Fig 2). The intra- and inter-assay coefficients of variability ranged from < 0.02–12.0% and 7.1–9.9% (Table 4), respectively. In all 12 experiments with a LOD of 10 copies, the analytical specificity and sensitivity were both 100%.

**Table 5:** Guinea worm qPCR inter-assay variability of standard curve dilution series (10^8^ to 10^1^) across 12 experiments (plates) at the University of Georgia. Nine experiments were conducted at the Southeastern Cooperative Wildlife Disease Study, and three were performed at other UGA laboratories.

Inter-assay variability (reproducibility)				
Calculated from 12 standard curve experiments				
	C_q_			
Standard dilution	Mean	SD	CV (%)	No. of wells positive (/36)
10^8	13.00	0.93	7.1	36
10^7	16.42	1.26	7.7	36
10^6	19.88	1.77	8.9	36
10^5	23.13	2.24	9.7	36
10^4	26.85	2.62	9.7	36
10^3	30.53	2.87	9.4	36
10^2	33.24	3.14	9.4	35
10^1	36.53	3.61	9.9	31

### Detection of Guinea worm DNA using qPCR and estimation of qPCR diagnostic parameters

Including the panels sent to external labs, all Guinea worm samples were detected as “positive” with no instances of “suspect” amplification; thus, the D_Sn_ of this assay is 100%. Non-target samples, including host DNA, non-*Dracunculus sp*. nematodes, *D*. *insignis*, and *D*. *ophidensis-like* DNA, did not amplify; thus, the D_Sp_ of this assay was calculated at 100%.

## Discussion

The primary objective of this work was to develop an assay that can provide rapid, genetic confirmation of suspect Guinea worm samples. The validation of a rapid molecular-based test that genetically confirms suspect Guinea worm samples allows for faster responses to reports of dracunculiasis and subsequently aids in implementing quick and effective control measures. Accordingly, the development and implementation of such a test are vital for advancing eradication and facilitating certification of dracunculiasis eradication.

During the initial stages of assay development, we faced challenges due to the lack of available sequence data for our chosen gene targets, particularly from non-target *Dracunculus* spp. and other relevant nematodes, which made it challenging to determine *in-situ* assay specificity. While the *cytb* assay was designed to exclude *D*. *lutrae* based on available mitochondrial sequences (Fig 1), there were no reliable *D*. *lutrae* samples available for testing. *Dracunculus lutrae* has been shown to exhibit high mitochondrial (*COI*) genetic diversity [40]; however, considering the lack of *D*. *lutrae cytb* sequences available on Genbank at the time of assay design, it is unknown if this gene is equally diverse. Obtaining and testing confirmed *D*. *lutrae* samples in the future would help to clarify this point. Regardless, *D*. *lutrae* is considered a host-specific parasite of North American river otters (*Lontra canadensis*) that has not been found outside the United States and Canada [40]. Accordingly, the likelihood of *D*. *lutrae* being present in Africa is extremely low. However, detection of Guinea worm from new or unusual hosts or in new locations should be supported by morphological and sequencing confirmation methods.

Thus far, we have determined that this assay has not amplified any known parasitic nematode species of animals found in Guinea worm-endemic areas. Other *Dracunculus* spp. currently documented in Africa besides Guinea worm include snake-infecting species such as *D*. *doi* on Madagascar and *D*. *dahomensis* in Benin [35]. Unfortunately, sequences for these snake-infecting *Dracunculus* spp. are lacking, but we obtained *D*. *ophidensis* samples (presumptive identification based on adult female morphology, host, location, and 18S rDNA sequencing) from North American snakes that were included in our assay validation [32,35]. These *D*. *ophidensis* samples did not amplify with this assay; thus, it is likely that other snake-infecting species would also be divergent enough from Guinea worm to not amplify; this has been seen with a previous 18S rRNA gene phylogenetic analysis, which included *D*. *oesophageus* from a European snake [41]. The *Dracunculus* sp. nematode (OPO28; GenBank accession no. MK085893.1) that most closely groups with the *Dracunculus* nematode recovered from a dog in Spain [26] also did not amplify. There are likely additional *Dracunculus* species not yet formally described [for example, the Vietnamese *Dracunculus* specimen recovered from a human; 42] for which there are no *cytb* sequences; therefore, continued validation of this assay with new *Dracunculus* spp. as they are discovered and verified would ensure the specificity of this assay.

The shift from infections only in humans to an increasing number of reported infections in domestic animals and wildlife complicates the eradication of Guinea worm [8,9,43]. The current system of confirming morphologically identified/suspect nematodes using Sanger sequencing is time-consuming and presents no opportunity to be adapted for use in the field. In contrast, the extremely high diagnostic sensitivity demonstrated by the GW qPCR assay described here lends strong support for its use in diagnostic laboratories, and it could be adapted for patient-side use through field-deployable qPCR technologies. Importantly, this assay was developed and validated using sections of worms or individual larvae. Additional validation would be needed should this assay be used to detect larvae within water samples/copepods or fish or frog tissues, as we did not evaluate the impacts of sampling protocols or how the number of copepods within a sample or the amount of tissue present would impact the sensitivity of the assay.

This validated qPCR assay for the species confirmation of Guinea worm-suspect samples will aid the Guinea worm eradication efforts by increasing the rapidity of diagnosis and, therefore, implementation of control measures. In the future, this assay could be adapted for use with field-deployable real-time PCR technology to further advance rapid Guinea worm species confirmation in Guinea worm endemic areas. For example, this could help obtain a preliminary diagnosis of suspect worms of new regions or hosts, or to investigate the role of transport and paratenic hosts [20–22,36,44]. A field-deployable assay would further decrease the time from sample collection to species confirmation. Implementation of this highly sensitive and specific assay by Guinea worm diagnosticians will be extremely beneficial in the fight against Guinea worm disease.

## Supporting information

S1 Methods The mitochondrial cytochrome *c* oxidase III (*cox3*) gene was tested as a possible target site for designing a qPCR protocol specific to *Dracunculus medinensis*. (DOCX)

S1 Table Primers and probe targeting the mitochondrial cytochrome *c* oxidase subunit 3 (*cox3*) gene of Guinea worm (*Dracunculus medinensis*) for qPCR amplification and development of a gBlock standard. (DOCX)
