# Supplementary material for: Development and validation of a quantitative PCR for the detection of Guinea worm (Dracunculus medinensis)
Source: PLoS Negl Trop Dis. 2022 Oct 7;16(10):e0010830. doi: 10.1371/journal.pntd.0010830 (PMC9581357; doi:10.1371/journal.pntd.0010830)
Supplement: S1 Methods — (DOCX) [file pntd.0010830.s001.docx]

SUPPLEMENTARY DATA

In addition to developing and validating the *cytb* gene assay, a qPCR assay was designed using the methods described above to target the *cox3* gene. In addition to 123 sequences from Guinea worm, four of the available *D. insignis* sequences partially aligned to the *cox3* sequence fragment. The primers and probes resulting from this *in silico* primer/probe design are reported in S1 Table. The standard curve production and estimation of qPCR assay parameters were performed according to the methods described above using a 229-bp sequence for positions 242-471; no adapter sequences were needed to generate the gBlock Gene Fragment. The 10-fold serial dilutions of the gBlock ranged from 1 x 10^8^ copies to 1 copy per reaction. Optimization using the standard curve resulted in an assay with high sensitivity (LOD = 1 x 10^1^ copies per reaction). Initial testing demonstrated no amplification of host DNA (n = 18) and consistent amplification of Guinea worm (n = 43). However, continued optimization and troubleshooting showed consistent nonspecific amplification of *D. insignis* (n = 4) so this assay was not pursued further. While the assay targeting the *cytb* gene was more specific and therefore more useful to the goals of the GWEP, the *cox3* assay did not react with non-Dracunculid nematode tissue samples (n = 11) and is potentially specific to the *Dracunculus* genus.
