# Supplementary material for: Development and validation of a quantitative PCR for the detection of Guinea worm (Dracunculus medinensis)
Source: PLoS Negl Trop Dis. 2022 Oct 7;16(10):e0010830. doi: 10.1371/journal.pntd.0010830 (PMC9581357; doi:10.1371/journal.pntd.0010830)
Supplement: S1 Table — (DOCX) [file pntd.0010830.s002.docx]

**S1 Table. Primers and probe targeting the mitochondrial cytochrome *c* oxidase subunit 3 (*cox3*) gene of Guinea worm (*Dracunculus medinensis*) for qPCR amplification and development of a gBlock standard.**

| Primer | Sequence (5'→3') | Gene Position  (Guinea worm) | Length (bases) | Est. Tm (℃)* | GC Content (%) | Amplicon Size (bp) |
| --- | --- | --- | --- | --- | --- | --- |
| F | TTTGATTCTTCTTTGGTTCC | 292-312 | 20 | 57, 58.2 | 35 | 130 |
| R | AACAGTAACAGCACTACTC | 421-402 | 19 | 57, 48.7 | 42.1 |  |
| P | FAM-ACCTAAAGGACACCAAGACATACCCAAC-BHQ | 349-321 | 28 | 68, 69.1 | 46 |  |

*Two melting temperature estimates are provided. The first was calculated during the assay design by the PrimerQuest software. The second was provided by the manufacturer (MilliporeSigma).
